# The Impact in Older Women of Ovarian *FMR1* Genotypes and Sub-Genotypes on Ovarian Reserve

**DOI:** 10.1371/journal.pone.0033638

**Published:** 2012-03-16

**Authors:** Norbert Gleicher, Andrea Weghofer, Ann Kim, David H. Barad

**Affiliations:** 1 Center for Human Reproduction (CHR) and Foundation for Reproductive Medicine, New York, New York, United States of America; 2 Department of Gynecological Endocrinology and Reproductive Medicine, Medical University Vienna, Vienna, Austria; 3 Department of Epidemiology and Social Medicine and Department of Obstetrics, Gynecology and Women's Health, Albert Einstein College of Medicine, Bronx, New York, United States of America; Institut Jacques Monod, France

## Abstract

We recently associated ovarian *FMR1*genotypes and sub-genotypes with distinct ovarian aging patterns. How they impact older females is, however, unknown. We, therefore, investigated 217 consecutive first *in vitro* fertilization (IVF) cycles in women >40 assessing oocyte yields, stratified for better (anti-Müllerian hormone, AMH >1.05 ng/mL) or poorer (AMH≤1.05 ng/mL) functional reserve (FOR)). Mean age was 42.4±2.0 years, mean AMH 0.76±0.92 ng/mL and mean oocyte yield 5.3±5.4. Overall, and in women with better FOR, *FMR1* did not affect oocyte yields. With poorer FOR (AMH≤1.05 ng/mL) women with *het-norm/high*, however, demonstrated higher oocyte yields (5.0±3.8) than those with *het-norm/low* sub-genotype 3.1±2.5; P = 0.03), confirmed after log conversion. Known associated with low FOR at young age, *het-norm/high*, thus, appears to preserve FOR into older age, and both *het* sub-genotypes appear to expand female reproductive lifespan into opposite directions.

## Introduction

Based on a normal range of CGG polynucleotide repeats of 26 to 34 (median 30), we recently reported ovarian genotypes and sub-genotypes of the *FMR1* gene, so named because of their association with distinct ovarian aging patterns [1]–[3]. They have to be differentiated from previously reported *FMR1* genotypes, clinically widely utilized to define neuro-psychiatric risks, associated with triple CGGn expansions to traditional premutation and full mutation genotype ranges [4]. Expanded CGGn beyond 34 repeats would under the ovarian genotype nomenclature represent so-called *high* sub-genotypes of either heterozygous (*het*) or homozygous (*hom*) genotypes (see Materials and Methods for further detail). Probably because never before associated with pathology, traditional *FMR1* genotypes do not address abnormally low CGGn counts, though they have been included in the ovarian genotype nomenclature as *low* ovarian sub-genotypes, and have been associated not only with distinct ovarian aging patterns but also with increased autoimmune risk [1]–[3].

Especially the so-called *het-norm/low FMR1* sub-genotypes has been strongly associated with a specific ovarian aging pattern, characterized by abundant follicle recruitment and, therefore, excessive functional ovarian reserve (FOR) at young age, and also with autoimmunity [2]. These associations occur in all races/ethnicities, though with varying prevalence [3]. Because of very active follicle recruitment at young age, *het-norm/low* women rather quickly deplete their FOR, often demonstrating already at young ages abnormally diminished ovarian reserve [2].

In comparison to other *FMR1* genotypes and sub-genotypes, one, therefore, can view *het-norm/low* as moving a woman's functional reproductive peak towards younger ages. How ovarian *FMR1* genotypes and sub-genotypes affect ovarian reserve at older female age is still not well understood, and was subject of investigation in this study.

## Materials and Methods

Assuming identical ovarian aging patterns, women with different *FMR1* genotypes and sub-genotypes should demonstrate similar FOR at advanced ages. FOR can be assessed in various ways, but, excluding obvious biases, oocyte yields in association with in vitro fertilization (IVF), likely, best reflect FOR [5]. In addition, FOR can, of course, also be defined by follicle stimulating hormone (FSH) [6], anti-Müllerian hormone (AMH) [7] and antral follicle counts [8].

### Patients

We investigated 217 consecutive first IVF cycles in women above age 40 and assessed, as previously reported [1]–[3], oocyte yields in association with different *FMR1* genotypes and sub-genotypes. In brief, the CGGn was determined for each patients, utilizing different laboratories, depending on insurance coverage (Genzyme Analytical Services, Westborough MA; Quest Diagnostics, Lyndhurst, NJ; and LabCorp, Burlington, NC). We previously determined that all laboratories used identical techniques, and found results compatible. Results were reported as number of triple CGG repeats per allele.

Utilizing test results from all of these laboratories, we previously reported that in regards to ovarian function, the normal range of CGG repeats on *FMR1* is 26–34 (median 30) [1]. This median also corresponds to the reported switching point between positive and negative message, as well as peak translation [9].

Based on this normal range, a woman can be normal (*norm*) if both of her alleles are in normal range, can be heterozygous (*het*) if one allele is in and one out of range, and homozygous (*hom*) if both alleles are out of range [1]. *Het* and *hom* patients can be further sub-divided into sub-genotypes, depending whether their abnormal count allele is above (*high*) or below (*low*) normal range. *Het* women, thus, can be *het-norm/high* and *het-norm/low*; *hom* women can be *het-high/high*, *het-high/low* or *het-low/low* [2], [3]. Because, as here again confirmed, *hom* patients are apparently very rare amongst infertility patients, the very few *hom* patients in this patient population were excluded from consideration. This study, therefore, only reports on associations of *norm* and *het* genotypes on FOR at advanced female ages.

FOR was defined by levels of AMH, as repeatedly before reported [7], and by oocyte yields in routine IVF cycles. Only first IVF cycles were analyzed. Since all patients were above age 40 years, they at our center are automatically considered to suffer from diminished FOR. This means that all patients receive ovarian stimulation through a microdose agonist protocol, utilizing an FSH product (300–450 IU) and a human menopausal gonadotropin (hMG) product (150 IU) daily. Products from different manufacturers were prescribed, based on the patient's insurance requirements.

All patients were managed by two senior physicians (N.G. and D.H.B.) who in their respective patient outcomes, based on years of comparative quality control evaluations, do not differ in regards to numbers of oocytes retrieved or pregnancy rates in IVF cycles. Both physicians utilize identical patient protocols. Such differences are considered the principle cause for inaccuracies in utilizing oocyte yields as reflections of FOR [5].

As all patients suffered because of their age from low FOR, they, based on AMH levels, were sub-divided into those with poorer pregnancy prognosis (AMH<1.05 ng/mL and better pregnancy chances (AMH≥1.05 ng/mL). We previously reported that this AMH cut off, at all ages, discriminates between lower and higher pregnancy chances in women with low FOR [10].

All women with low FOR are at our center automatically supplemented with dehydroepiandrosterone (DHEA) (25 mg TID) for at least six weeks prior to IVF cycle start [11]. Here presented cycle outcome data are, therefore, not necessarily applicable to other women with low FOR, who are not DHEA supplemented.

### Statistical analysis

*FMR1* genotypes are reported as counts (%), while all other variables are reported as means ± standard deviation. Cancelled cycles (n = 20) are counted as 0 eggs.

Differences between normally distributed variables were tested with analysis of variance. Differences between groups of variables not conforming to normality were tested for with the Mann-Whitney test. A P of <0.05 was considered statistically significant. Statistical analyses were performed using the Statistical Package for Social Science version 18.0 (SPSS Inc., Chicago, IL)

### Institutional Review Board

Reported data were extracted from the center's anonymized electronic research database, and, where necessary, supplemented by individual chart reviews. All of the center's patients sign at time of initial presentation an informed consent, which allows the study of their medical records for research purposes as long as the patient's anonymity is preserved and the medical record remains confidential. If both conditions are met, studies require only expedited review under the center's Institutional Review Board (IRB) rules. Both conditions were met for this study. In addition, all of the center's research staff is in writing committed to confidentiality under federal HIPAA rules.

## Results

The 217 women undergoing their first IVF cycles were on average age 42.4±2.0 years old. Their mean AMH was 0.76±0.92 ng/mL, and mean oocyte yield was 5.3±5.4 (Table 1).

**Table 1:** Patient characteristics.

		*FMR1* genotype and sub-genotype		
	Total	*norm*	*het-norm/high*	*het-norm/low*
Patients/cycles	217	126 (58.1%)	35 (16.1%)	56 (25.8%)
Age (years)	42.4±2.0	42.5±2.0	41.9±2.0	42.4±2.1
AMH (ng/mL)	0.76±0.92	0.81±0.89	0.89±1.0	0.84±0.91
Oocyte yield (n)	5.3±5.4	5.2±5.2	6.1±6.1	5.0±5.3

**Figure 1:**
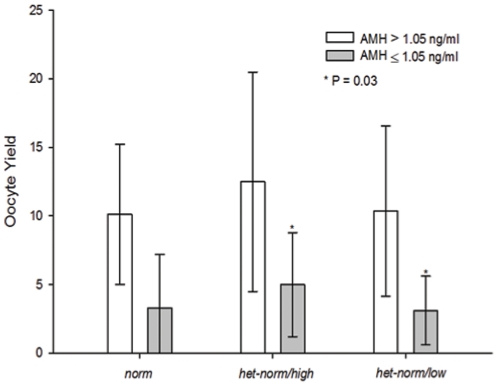
Oocyte yields in association with *FMR1* genotypes and sub-genotypes. Here presented data exclude 20 cancelled cycles, and, therefore, represent 23 *het norm/high* and 33 *het-norm/low* women for statistical comparison. Including 9 cancelled cycles in these two sub-genotypes, the comparison maintains significance (P = 0.03). It also maintains significance using the independent samples Mann-Whitney U test (data not shown).

None of the parameters reported in this table differed significantly between the different FMR1 genotypes and sub-genotypes. Once patients were, however, separated into poorer (AMH≤1.05 ng/mL) and better FOR (AMH>1.05 ng/mL), differences became apparent (Figure 1).

In assessing distribution of *FMR1* genotypes and sub-genotypes, 126/217 (58.1%) were *norm*, 35 (16.1%) were *het-norm/high*, and 56 (25.8%) were *het-norm/low*. In order, *norm*, *het-norm/high* and *het-norm/low* patients produced 5.2±5.2, 6.1±6.1 and 5.0±5.3 oocytes, a not significant difference; and in same order, AMH values were 0.81±0.89 ng/mL, 0.89±1.0 ng/mL and 0.84±0.91 ng/mL, also a non-significant difference.

Once patients were, however, assessed based on degree of diminished FOR, differences became apparent: While oocyte yields still did not differ among *FMR1* genotypes and sub-genotypes in women with better ovarian reserve (AMH≥1.05 ng/mL), women with poorer FOR (AMH<1.05 ng/mL) demonstrated significantly higher oocyte yields with *het norm/high FMR1* sub-genotype (5.0±3.8) than with *het-norm/low* sub-genotype (3.1±2.5, P = 0.03; Figure 1). Log-conversion, and adjustment for age maintained significance.

As Figure 1 demonstrates, *norm* patients fell in their oocyte yields between the two *het* sub-genotypes.

## Discussion

Here presented study, once again, demonstrates that ovarian aging patterns differ, depending on ovarian *FMR1* genotypes and sub-genotypes. We previously in cross-sectional studies demonstrated distinct differences between *norm* and *het* ovarian genotypes [1] and between *het* sub-genotypes [2], [3]. Because *hom* genotypes and sub-genotypes are rare, they have not, yet, been well defined.

These ovarian genotypes and sub-genotypes have to be distinguished from classical *FMR1* genotypes, which for decades have been used to, primarily, define neuro-psychiatric risks with premutation range (55–200 CGG repeats) and full mutation (fragile X syndrome) genotypes (>200 CGG repeats) [4].

As the only not neuro-psychiatric complication of the classical premutation genotype, the *FMR1* gene has for decades also been known associated with increased risk for premature ovarian failure (POF) [12], now also frequently called primary ovarian insufficiency (POI). It was this association that made us suspect that the gene may, possibly, also have ovarian functions [11].

This suspicion was further strengthened when, upon reviewing the published literature, we came across a study of CGG distribution in a general population by Fu et al, which demonstrated a very significant distribution peak at 29–30 repeats [13]. Moreover, Chen et al had reported that 30 repeats represented the switching point for positive message and peak translation for the gene product, fragile X mental retardation protein [9].

Utilizing our own infertile patients, we determined a normal range of 26–34 CGG repeats in regards to the gene's ovarian function, with 30 repeats representing the median [1]. Range and median were identified as identical in different ethnicities/races [14]. Based on this normal range, we then defined patients as *norm*, *het* or *hom*, depending whether both, one or none of their two alleles were in normal range [1]. Moreover, *het* and *hom* genotypes could then be sub-divided, based on whether abnormal alleles were above (*high*) or below (*low*) the normal range [2], [3].This revealed distinct differences between *het-norm/high* and *het-norm/low* sub-genotypes [2]: *Het-norm low* is at young age associated with a polycystic ovary (PCO)-like ovarian phenotype, which rapidly depleted its ovarian reserve, at mid-age often already leading to prematurely diminished FOR. The sub-genotype was also associated with autoimmunity but this association, remarkably, reached approximately 90 percent when the sub-genotype was associated with a PCO-like ovarian phenotype. In contrast, the *het-norm/high* sub-genotype proved almost protective for autoimmunity, with only, barely, a 10 percent prevalence [2].

A further discussion of autoimmune findings exceeds the framework of this manuscript. The reviewer is directed to another, recent publication [15]. Clinically most relevant for infertility practice, *het-norm/low* was also associated with significantly diminished IVF pregnancy rates in comparison to *norm* women, with *het-norm/high* patients falling in the middle [2].

Trying to understand the possible mechanisms behind this difference in pregnancy rates, we confirmed their association with the different ovarian genotypes and sub-genotypes in different ethnicities/races, which demonstrate greatly varying distributions of these genotypes and sub-genotypes, and, accordingly, also different IVF pregnancy chances and autoimmune prevalence [3].

Here, in this study, we attempted to better define the impact of female age. By investigating 217 consecutive first IVF cycles in women above age 40 years, the study offers a robust sample size of older women, seeking fertility treatment. Women at that age, universally, can be considered to suffer from low FOR [6]. At our center they, therefore, are routinely supplemented for at least six weeks with DHEA before an IVF cycle is initiated [11]. Here presented data for this reason have to be interpreted with caution since they may not be applicable to non-supplemented IVF cycles in older women.

We previously reported that an AMH level of 1.05 ng/mL in women with diminished FOR defines at all ages a cut off between better and poorer IVF pregnancy chances [10]. Here presented results expand on these observations, as they demonstrate that in older women, above age 40 years, their *FMR1* genotypes and sub-genotypes matter little in regards to oocyte yields in IVF, as long as they still have minimally fair ovarian reserve, defined as an AMH of ≥1.05 ng/mL. Since pregnancy chances usually follow oocyte yields, it appears likely that pregnancy chances also will not differ with different *FMR1* genotypes and sub-genotypes, as long as AMH levels are ≥1.05 ng/mL, though this fact remains to be confirmed, as here studied cycle volume was too small to assess pregnancy outcomes.

Below AMH of 1.05 ng/mL, *FMR1* genotypes and sub-genotypes, however, suddenly do to a significant degree matter: It does not surprise that the *het-norm/low* sub-genotype, once again, is associated with low oocyte yields, therefore, low FOR and, likely, lower pregnancy chances with IVF. We previously reported lower IVF pregnancy chances in association with IVF [3]. The here observed unusually excellent performance of older women with *het-norm/high* sub-genotypes, despite very poor FOR, however, does somewhat surprise.

In a general infertility population, IVF pregnancy rates with *het-norm/high* sub-genotype were observed approximately half way between *norm* (best rates) and *het-norm/low* patients (worst rates) [2]. Here, amongst women above age 40 years, oocyte yields in *het-norm/high* women outperformed both, *norm* (second-best) and *het-norm/low* patients (worst, Figure 1).

This represents a second observed instance in which the two ovarian *het FMR1* sub-genotypes perform at opposing clinical extremes: A similar opposing association between the two *het* sub-genotypes was previously seen in definition of an autoimmune phenotype, when *het-norm-high* appeared protective and *het-norm/low* strongly enhancing for risk towards autoimmunity [2].

Why two sub-genotypes of the same genotype would reflect such opposing phenotypes allows for speculation: One could assume that the original *FMR1* gene only contained what, now, represents the *norm* genotype. This genotype favors reproduction at relatively young age, when, due to robust recruitment of primordial follicles, FOR is high. Because of active recruitment, women with the *norm* genotype, therefore, deplete their FOR a relatively young ages [1]. Their reproductive lifespan, consequently, would have to be homogenous and rather short.

Preservation of the species would, likely, favor less homogenous and, therefore, overall longer reproductive lifespans. One, therefore, could further speculate that evolution would favor gene mutations, which within a community allow for diversity of reproductive lifespans, some extending towards younger and others towards older age.

Here presented data are potentially supportive of the hypothesis that, amongst all *FMR1* genotypes and sub-genotypes, the *het-norm/high* sub-genotype appears to preserve FOR into advanced female age the best. In contrast, as noted earlier, *het-norm/low* is associated with rapid depletion of FOR at young age and prematurely low FOR [2].

Both of these sub-genotypes could, thus, be seen as mutations from an originally *norm* range *FMR1* gene, with the evolutionary goal to expand the community's reproductive lifespan into both directions, towards younger age with *het/norm/low* mutations, and towards older age with *het-norm/high* sub-genotypes.

While, thus, hypothetically advantageous to preservation of the species, both mutations also result in highly disadvantageous phenotypes to the human species: As already noted before, abnormally low CGG counts, represented by *het-norm/low*, significantly increase the risk towards autoimmunity [2], [3], potentially explaining why women carry a many-fold higher risk [16]. *Het-norm/high FMR1* mutations, by veering into CGG repeats above *norm*, create potential neuro-psychiatric risks, if expansion sizes reach classical premutation and full mutation ranges [4], [12]. This study, thus, offers an expansive hypothetical view of the importance of the *FMR1* gene, which until recently almost exclusively was only known for its neuro-psychiatric risks.
